# Modulation of Cytokine Production by Drugs with Antiepileptic or Mood Stabilizer Properties in Anti-CD3- and Anti-CD40-Stimulated Blood *In Vitro*

**DOI:** 10.1155/2014/806162

**Published:** 2014-03-16

**Authors:** Hubertus Himmerich, Stefanie Bartsch, Hajo Hamer, Roland Mergl, Jeremias Schönherr, Charlotte Petersein, Alexander Munzer, Kenneth Clifford Kirkby, Katrin Bauer, Ulrich Sack

**Affiliations:** ^1^Department of Psychiatry, University of Leipzig, Semmelweisstr. 10, 04103 Leipzig, Germany; ^2^Epilepsy Center, Department of Neurology, University Hospital Erlangen, 91054 Erlangen, Germany; ^3^Department of Psychiatry, University of Tasmania, Hobart, TAS 7001, Australia; ^4^Institute of Immunology, University of Leipzig, Johannisallee 30, 04103 Leipzig, Germany

## Abstract

Increased cytokine production possibly due to oxidative stress has repeatedly been shown to play a pivotal role in the pathophysiology of epilepsy and bipolar disorder. Recent *in vitro* and animal studies of valproic acid (VPA) report antioxidative and anti-inflammatory properties, and suppression of interleukin (IL)-6 and tumor necrosis factor (TNF)-**α**. We tested the effect of drugs with antiepileptic or mood stabilizer properties, namely, primidone (PRM), carbamazepine (CBZ), levetiracetam (LEV), lamotrigine (LTG), VPA, oxcarbazepine (OXC), topiramate (TPM), phenobarbital (PB), and lithium on the production of the following cytokines *in vitro*: interleukin (IL)-1**β**, IL-2, IL-4, IL-6, IL-17, IL-22, and TNF-**α**. We performed a whole blood assay with stimulated blood of 14 healthy female subjects. Anti-human CD3 monoclonal antibody OKT3, combined with 5C3 antibody against CD40, was used as stimulant. We found a significant reduction of IL-1 and IL-2 levels with all tested drugs other than lithium in the CD3/5C3-stimulated blood; VPA led to a decrease in IL-1**β**, IL-2, IL-4, IL-6, IL-17, and TNF-**α** production, which substantiates and adds knowledge to current hypotheses on VPA's anti-inflammatory properties.

## 1. Introduction

Immunological processes play a role in the pathophysiology of a variety of brain diseases such as infections, autoimmune, or neurodegenerative diseases and psychiatric disorders [1–10]. Specifically, changes in the immune system have been implicated in the pathophysiology of bipolar disorder and some types of epilepsies [11–13].

One possible cause of cytokine changes in epilepsy and bipolar disorder is oxidative stress. Oxidative stress is a state of imbalance in the production of reactive oxygen species (ROS) and nitrogen [14], which increases production of proinflammatory cytokines such as interleukin (IL)-1, IL-6, and tumor necrosis factor-*α* (TNF-*α*) [15–19]. The genetic make-up of the defense system against oxidative stress, for example, genetic variants of the superoxide dismutase gene, also influences cytokine production [20]. Increasing evidence indicates that oxidative stress can play a role in a wide range of neurological and psychiatric disorders, including epilepsy and affective disorders [21–24].

Proinflammatory cytokines have also been shown to lead to oxidative stress by producing reactive oxygen species [25, 26]. Besides oxidative stress, cytokines can be altered due to genetic predisposition, psychosocial stress, sleep disturbance, inadequate nutrition, and changes in cellular components of the immune system [27–30].

For epilepsy and bipolar disorder, overlapping results regarding the cytokine system have been reported, namely, alterations of IL-1**β**, IL-2, IL-4, IL-6, and TNF-*α* [12, 31–34]. Of these, data regarding IL-2 and IL-4 is limited and the few studies do not show consistent results. Also, the involvement of IL-17 and IL-22 in the pathogenesis of epilepsy or bipolar disorder has not been investigated, although they play important roles in inflammatory immune responses [35–38]. Bipolar disorder and epilepsy not only share immunological abnormalities; some antiepileptic drugs are also used to treat bipolar disorder. Valproic acid (VPA), carbamazepine (CBZ), and lamotrigine (LTG) are antiepileptic drugs (AEDs) which are evidence-based treatments for bipolar disorder. There are also indications of therapeutic potential for the AEDs oxcarbazepine (OXC), topiramate (TPM), and levetiracetam (LEV) in bipolar disorder [39].

*In vitro* and* in vivo* experiments show that AEDs as well as mood stabilizers such as VPA and lithium can affect cytokine levels. In patients with epilepsy, CBZ, VPA and phenytoin were reported to lead to elevated levels of IL-1*β*, IL-2, IL-5, IL-6, and TNF-*α* [40, 41].* In vitro*, however, CBZ, VPA, and phenobarbital (PB) were reported to inhibit the production of IL-2, IL-4, IL-6, and TNF-*α* [40–42]. In patients with affective disorders, CBZ and lithium led to increased plasma concentrations of TNF-*α* and its soluble receptors sTNF-R p55 and p75 [43]. The discrepancy of results of* in vitro* versus* in vivo* experiments enjoins us to interpret the results of* in vitro* experiments with caution. Nevertheless, to better understand mechanisms of action and of side effects, it is important to know effects of psychopharmacological agents on different tissues such as blood, liver, or brain tissue.

A relevant line of research in this context is that, in depression and bipolar disorder, the stimulated* in vitro* production of cytokines has been shown to differ in patients versus controls and to change during successful therapy [44–46]. In recent research, we systematically measured levels of IL-1**β**, IL-2, IL-4, IL-6, IL-17, IL-22, and TNF-*α* in toxic shock syndrome toxin-1 (TSST-1-) stimulated blood supplemented with PRM, CBZ, LEV, LTG, VPA, OXC, TPM, PB, or lithium in a whole blood assay [47]. In this study, we found that IL-1**β** production was significantly decreased by PRM, CBZ, LEV, LTG, OXC, PB, and lithium. IL-2 significantly decreased by PRM, CBZ, LEV, LTG, VPA, OXC, TPM, and PB. IL-22 significantly increased by PRM, CBZ, LEV, OXC, TPM, and lithium and decreased by VPA. TNF-*α* production significantly decreased under all applied drugs [47]. The immunological stimulant TSST-1 used in this study leads to nonspecific binding of major histocompatibility complex class II (MHC II) with T cell receptors, resulting in polyclonal T cell activation, stimulation of mononuclear cells, and increased cytokine production [48, 49].

In the present study, we aimed to delineate the influence of these drugs on cytokine production by T and B cells. Therefore, we used specific stimulators, known to induce cytokine production in T and B cells. Murine anti-human CD3 monoclonal antibody OKT3 (muromonab-CD3) binds to the T cell receptor CD3 complex and is an established T cell activator [50]. 5C3 monoclonal antibody which reacts with human CD40 is reported to activate B cells in* in vitro* functional assays [51]. CD40 is a costimulatory protein found on antigen presenting cells and is required for their activation [52, 53]. It is known that activation of CD40 stimulates ROS production by an NADPH oxidase. CD40 receptor stimulation also increases phosphoinositide 3-kinase (PI3K) activity. PI3K, in turn, activates GTPase Rac1 and increases ROS generation such as H_2_O_2_ and O_2_^•−^ [54] which might contribute to cytokine activation. Additionally, several other mechanisms have been proposed by which CD40 leads to cytokine production, such as protein kinase B (Akt) and nuclear factor (NF)-kappa B (NF-*κ*B) signaling pathways [55].

## 2. Methods and Material

*Subjects.* 14 healthy female subjects between 22 and 47 years of age (mean: 29 + 6.4 (SD) years). Exclusion criteria were used of illegal drugs or regular alcohol consumption, presence of any immunological, infectious or endocrinological disorder, and a history of psychiatric disorder from an interview by a psychiatrist using the Structured Clinical Interview for DSM-IV (SKID-I; German) [56].

*Experimental Procedure.* The whole blood assay was performed as described previously [57–59]. Blood was taken from all subjects once with a heparin-monovette (Sarstedt, Nürtingen, Germany) and cultured in a whole blood assay within 1–2 h after blood collection. Cell concentration was adjusted at 3–4 × 109 cells/L using RPMI 1640 medium (Biochrom, Berlin, Germany). Subsequently, 100 *μ*L of this blood plus RPMI solution was introduced into a tube and mixed with 100 *μ*L pure psychopharmacological substance plus RPMI, resulting in a final cell concentration of 1.5–2 × 109 cells/L.

The final concentration of each AED in this mixture was chosen as to the upper reference value of the therapeutic range of the local clinical-chemical laboratory [60]. The concentration of lithium was chosen in accordance with the AGNP-TDM expert group consensus guidelines: therapeutic drug monitoring in psychiatry [61]. We used the following concentrations: PRM: 12 *μ*g/mL, CBZ: 10 *μ*g/mL, LEV: 90 *μ*g/mL, LTG: 12 *μ*g/mL, VPA: 100 *μ*g/mL, OXC: 30 *μ*g/mL, TPM: 25 *μ*g/mL, PB: 40 *μ*g/mL, and lithium: 1.2 mmol/L. We will subsequently refer to these concentrations as “1-fold.” We additionally tested 2-fold these concentrations, that is, 24 *μ*g/mL, CBZ: 20 *μ*g/mL, LEV: 180 *μ*g/mL, LTG 24 *μ*g/mL, VPA: 200 *μ*g/mL, OXC: 60 *μ*g/mL, TPM: 50 *μ*g/mL, PB: 80 *μ*g/mL, and lithium: 2.4 mmol/L.

The control condition was a tube likewise filled with blood and medium, without any psychopharmacological substance. According to the design of this experiment, we prepared 20 samples, one per tube, from the blood of each participant: one tube as unstimulated control condition, one as stimulated control condition, and 18 tubes under stimulated conditions with one of the nine drugs in 2 different concentrations (1-fold and 2-fold concentration). For induction of all cytokines, we used 100 ng/mL OKT3 plus 100 ng/mL 5C3 (OKT3/5C3).

As we investigated the blood of 14 donors, we had 14 times 20 equals 280 samples in total. Pure substances of the drugs were obtained from Sigma-Aldrich Laborchemikalien GmbH (Seelze, Germany). All tubes were covered and samples incubated in an atmosphere of 5% CO2 and 37°C for 48 h. Cell-free supernatants were harvested after incubation and stored at minus 70°C.

For quantification of cytokines IL-1**β**, IL-2, IL-4, IL-6, IL-17, and TNF-*α*, we used bead array flow cytometry (FACSArray Bioanalyzer, BD Biosciences, Franklin Lakes, NJ, USA). IL-22 was determined using a human IL-22 DuoSet Elisa (R&D Systems Europe, Abingdon, UK).

*Statistical Analysis.* Because of the nonnormal distribution and small number of data points, all comparisons between cytokine concentrations were undertaken with nonparametric paired Wilcoxon tests. Due to the exploratory nature of this study, an uncorrected *P* value below 0.05 was considered significant.

## 3. Results

*General Findings.* Stimulation significantly increased the concentration of all cytokines (IL-1**β**, IL-2, IL-4, IL-6, IL-17, IL-22, and TNF-*α*); see Table 1 for descriptive statistics of cytokine levels and for the comparison between unstimulated and OKT3/5C3-stimulated blood. Without stimulation, cytokines were not measurable in most samples. For example, IL-22 levels were below the detection level in 12 of 14 unstimulated samples (*N* = 2; see Table 1), whereas stimulation with OKT3/5C3 rendered IL-22 detectable in most cases. However, the number of cases *N* = 2 of measurable IL-22 levels in the unstimulated samples was too small to obtain a significant difference in the Wilcoxon test when comparing stimulated and unstimulated IL-22 levels.

**Figure 1:**
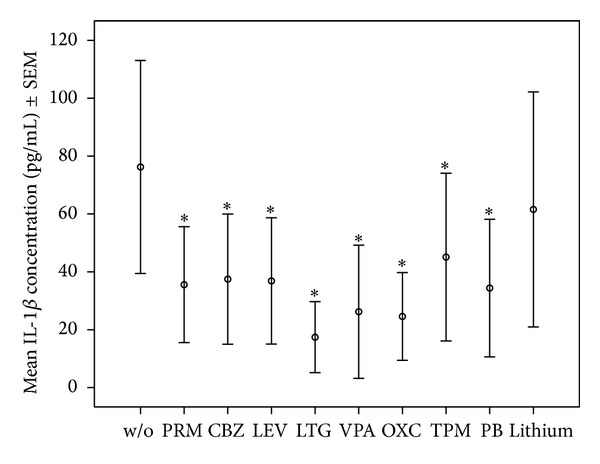
Mean ± SEM of IL-1**β** concentrations in OKT3/5C3-stimulated whole blood assay without or with mood stabilizers or AEDs at 1-fold concentration (PRM: 12 *μ*g/mL, CBZ: 10 *μ*g/mL, LEV: 90 *μ*g/mL, LTG: 12 *μ*g/mL, VPA: 100 *μ*g/mL, OXC: 30 *μ*g/mL, TPM: 25 *μ*g/mL, PB: 40 *μ*g/mL, and lithium: 1.2 mmol/L). *Significant difference between cytokine values in OKT3/5C3-stimulated blood and OKT3/5C3-stimulated blood with supplementation of the listed drugs.

**Figure 2:**
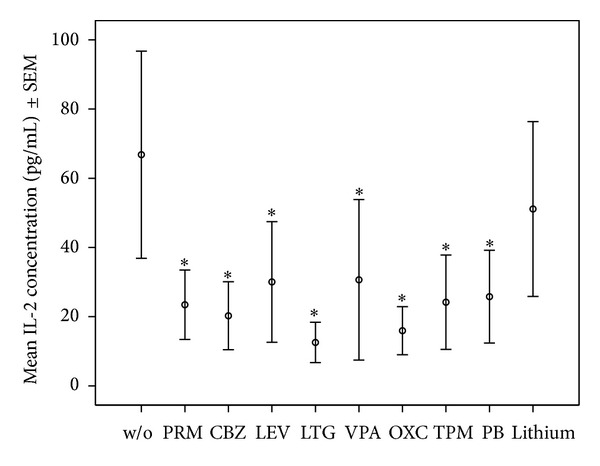
Mean ± SEM of IL-2 concentrations in OKT3/5C3-stimulated whole blood assay without or with mood stabilizers or AEDs at 1-fold concentration. *Significant difference between cytokine values in OKT3/5C3-stimulated blood and OKT3/5C3-stimulated blood with supplementation of the listed drugs.

**Figure 3:**
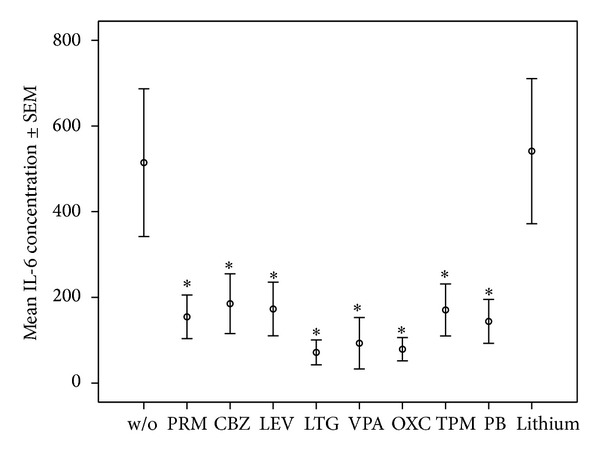
Mean ± SEM of IL-6 concentrations in OKT3/5C3-stimulated whole blood assay without or with mood stabilizers or AEDs at 1-fold concentration. *Significant difference between cytokine values in OKT3/5C3-stimulated blood and OKT3/5C3-stimulated blood with supplementation of the listed drugs.

**Figure 4:**
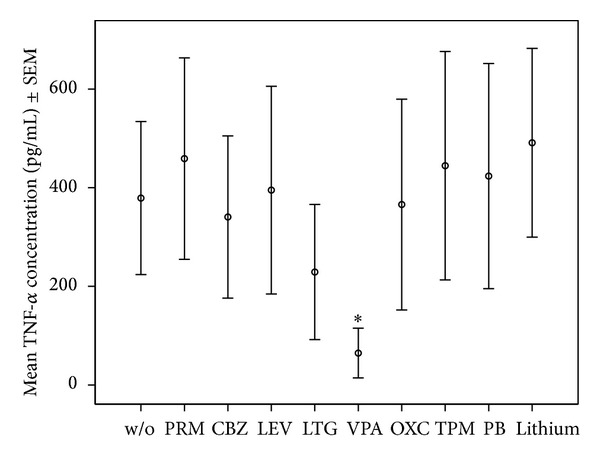
Mean and ± SEM of TNF-*α* concentrations in OKT3/5C3-stimulated whole blood assay without or with mood stabilizers or AEDs at 1-fold concentration. *Significant difference between cytokine values in OKT3/5C3-stimulated blood and OKT3/5C3-stimulated blood with supplementation of the listed drugs.

*Specific Findings.* Details of median and quartiles of measured cytokines are shown in Table 1. Means ± standard error of the mean (SEM) of IL-1**β**, IL-2, IL-6; and TNF-*α* for assays with the 1-fold drug concentration is shown in Figures 1, 2, 3, and 4.

We focus in this section mainly on those significant findings seen at both applied concentrations, assuming these findings to have the highest consistency. IL-1**β** production was significantly lowered by most AEDs, namely, PRM, CBZ, LEV, LTG, OXC, VPA, and PB at both applied concentrations, but not lithium in any concentration. IL-2 production decreased significantly under PRM, CBZ, LEV, LTG, VPA, OXC, and TPM in both concentrations, whereas IL-2 increased significantly under lithium at 2-fold concentration. VPA and LTG reduced IL-4 levels consistently across the two applied concentrations; IL-6 levels decreased significantly under PRM, CBZ, LEV, LTG, VPA, OXC, and TPM at both concentrations and PB at 1-fold concentration, and not under lithium. IL-17 decreased significantly under LTG and VPA at both concentrations and increased under lithium. IL-22 levels were significantly increased by lithium at 2-fold concentration. Finally, TNF-*α* production decreased significantly only under VPA at both applied concentrations.

Some immunomodulatory effects of the tested drugs were dose dependent (see Table 1). However, the differences in cytokine production between the two tested drug concentrations were not systematically significant.

## 4. Discussion

In this* in vitro* paradigm, blood cells were stimulated by OKT3 and 5C3 antibodies to enhance the modulatory effects of AEDs and lithium on cytokine production. The main findings were that the significant reduction of IL-1 and IL-2 levels was made by most of the tested drugs but not lithium and that VPA leads to a decrease in IL-1**β**, IL-2, IL-4, IL-6, IL-17, and TNF-*α* production. No other antiepileptic drug or mood stabilizer led to such a general decrease in cytokine production.

In bipolar disorders as well as in epilepsy and febrile seizures, IL-1**β** levels have been reported to be increased. IL-1**β** has been hypothesized to contribute to the pathogenesis of epilepsy, and anti-IL-1**β** medication has been hypothesized to have therapeutic potential as AED [13, 32, 62, 63]. Therefore, the decrease in IL-1**β** production may be a complementary mechanism by which AEDs exert their antiepileptic action.

Our findings that all AEDs reduced IL-2 production in a whole blood assay are in line with previous studies which showed that CBZ [41], PB [42] of PRM, LEV, LTG, VPA, OXC, and TPM [47] inhibit stimulated IL-2 production* in vitro*. This finding may also be relevant for the action of antiepileptic drugs in the brain, because IL-2 is epileptogenic, producing EEG alterations after intracerebroventricular administration such as single spikes, polyspikes, or spike waves [64, 65].

One possible explanation how AEDs and mood stabilizers influence immune cells could be the modulation of ion channels. Immune cells express these channels, and they are important for their function. Specific lymphocyte functions such as lymphocyte development, selection, differentiation, invasive capacity, cytotoxicity, T cell receptor activation, and cytokine production all depend on ion-conducting channels for sodium, potassium, calcium, and chloride [66–70].

Not only in lymphocytes but also in macrophages sodium channels serve important functions. In macrophages they are necessary for organelle polarization and are therefore expressed in endosomes and phagolysosomes to regulate phagocytosis [71]. Dysfunction of these channels in macrophages is hypothesized to contribute to a broad spectrum of health problems ranging from an attenuated defense against mycobacteria [72] to the development of multiple sclerosis lesions [71].

As mentioned above, some AEDs (VPA, PB, and TPM) act on the GABA system. In recent years, GABA has been shown to act as an immunomodulatory molecule and appears to modulate a wide variety of functional properties of the cells including cell proliferation, cytokine secretion, phagocytic activity, and chemotaxis [73–76]. GABA receptors seem to be important, for example, for T lymphocytes, as different subtypes of GABA receptors are expressed in human, mouse, and rat T lymphocytes [77]. One has to bear in mind that the GABA-A receptor is an ionotropic receptor which selectively conducts chloride ions through its pore, resulting in hyperpolarization of a cell.

In the present study, VPA led to decreased production of various cytokines, namely, IL-1**β**, IL-2, IL-4, IL-6, IL-17, and TNF-*α*. It has already been shown that VPA suppresses lipopolysaccharide-induced production of TNF-*α* and IL-6* in vitro* [78, 79]. It is also reported that VPA inhibits the ischemia-induced nuclear translocation of nuclear factor-*κ*B (NF*κ*B) activation and matrix metalloproteinase 9 production* in vivo* and has protective effects against various types of ischemia and reperfusion injury as well as inflammatory diseases [80–84].

In a very recent and, in our opinion, methodologically rigorous study regarding the influence of VPA on ischemic, inflammatory, and oxidative damage in rats, Suda et al. [85] explored the effect of VPA on experimental ischemic stroke and on myeloperoxidase (MPO), microglia (Iba1), 4-hydroxy-2-nonenal (4-HNE), and 8-hydroxy-deoxyguanosine (8-OHdG). MPO produces hypochlorous acid (HOCl) from H_2_O_2_ and chloride anion (Cl^−^). 4-HNE is a product and mediator of oxidative stress [86]. 8-OHdG is a marker of oxidative DNA damage which has been shown to be increased, for example, in the urine of patients with depression [87].

Suda et al. found that VPA significantly reduced infarct volume and improved neurological deficit in rats under oxidative stress. Moreover, VPA significantly reduced MPO-positive cells, Iba1-positive cells, 4-HNE-positive cells, and 8-OHdG-positive cells compared with vehicle in the ischemic boundary zone. They concluded from their results that VPA has anti-inflammatory as well as antioxidative effects [85]. The inhibition of TNF-*α* production along with a decrease in MPO release due to VPA has also recently been found in a peritonitis paradigm in mice [88]. These findings of antioxidative and anti-inflammatory properties of VPA are consistent with our* in vitro* results of a decrease in cytokine production.

This study only included young female subjects and does not permit generalization to male subjects or other age groups. We did not control for the menstrual cycle as a possible confounding factor. However, a systematic bias is unlikely.

In previous studies, we used TSST-1 for stimulation to enhance the modulatory effects of different drugs on cytokine production [47, 59, 89]. TSST-1—as already explained in the introduction—is a staphylococcal-secreted exotoxin which leads to nonspecific binding of major histocompatibility complex class II with T cell receptors, resulting in T as well as B cell activation, stimulation of mononuclear cells, and increased cytokine production [48, 49, 90]. Thus, TSST-1 is a very reliable but supraphysiological immunological stimulator which may therefore be too strong to simulate blood cells in a clinically relevant manner. Hence, in the present study, we sought to stimulate only lymphocytes using OKT3 combined with 5C3 to influence CD3 and CD40. This approach has successfully been tested for investigating the effect of antidepressants on cytokine production* in vitro* [91]. But in further studies one should use either OKT3 or 5C3 to be able to separate T cell from B cell effects. However, in our previous study using TSST-1 for stimulation, we obtained similar results: IL-1*β* production was significantly decreased by PRM, CBZ, LEV, LTG, OXC, PB, and lithium, and IL-2 was significantly decreased by PRM, CBZ, LEV, LTG, VPA, OXC, TPM, and PB [47]. Therefore, one can conclude that the results regarding IL-1**β** and IL-2 show consistency across two different methods.

Another limitation of our study is that the reported effects shown in this* in vitro* experiment may not be therapeutically relevant for all patients, because most epileptic or bipolar patients do not receive the maximum therapeutic dose. Therefore, it would be advisable for further studies to use lower drug doses too.

Besides IL-1**β**, IL-2, IL-4, IL-6, IL-17, IL-22, and TNF-*α*, several other cytokines such as IL-10, interferon-*γ* (IFN-*γ*), transforming growth factor (TGF)-*β*, erythropoietin (EPO), cytokine receptors such as the TNF-*α* receptors TNF-R p55 and TNF-R p75, and cytokine receptor antagonists such as the IL-1 receptor antagonist (IL-1ra) have been implicated in the pathophysiology of psychiatric and neurological disorders [2, 92, 93]. Therefore, we may have missed effects of AEDs and mood stabilizers on one of these important cytokines.

We did not measure markers of cell death in the reported experiments. Therefore, we can not rule out that cytotoxicity may have contributed to modification of cytokine production due to the tested drugs.

In the statistical analysis we have reported all significant effects at a* P* level of less than 0.05. We did not have access to previous comparable empirical results of experiments using anti-CD3- and anti-CD40-stimulated blood we did not have access to previous comparable empirical results of experiments using anti-CD3- and anti-CD40-stimulated blood. Therefore, we did not have any data for a prospective power analysis while planning this study.

In conclusion, we found significant reductions in IL-1 and IL-2 production by most of the AEDs and mood stabilizers but not lithium. The decrease in cytokine signaling may be a complementary mechanism of action of these drugs in the therapy of epilepsy and bipolar disorder. We also found reduction of IL-1**β**, IL-2, IL-4, IL-6, IL-17, and TNF-*α* release by VPA. These results provide supportive evidence for current hypotheses regarding VPA's anti-inflammatory and antioxidative properties.

## Figures and Tables

**Table 1:** Median, first (1. Qu), and third (3. Qu) quartile of cytokine levels in unstimulated blood, OKT3/5C3-stimulated blood, and OKT3/5C3-stimulated blood with mood stabilizers and AEDs at 1-fold (PRM: 12 *μ*g/mL, CBZ: 10 µg/mL, LEV: 90 *μ*g/mL, LTG: 12 *μ*g/mL, VPA: 100 *μ*g/mL, OXC: 30 *μ*g/mL, TPM: 25 *μ*g/mL, PB: 40 *μ*g/mL, and lithium: 1.2 mmol/L) and 2-fold concentration.

	IL-1*β* (pg/mL)								
	Median	1. Qu	3. Qu	*N*					
Unstimulated blood	0.01	0.00	0.07	14					
OKT3/5C3-stimulated blood^†^	23.47^†^	1.74	87.09	13					

Concentration	1-fold				2-fold				Sig.
	Median	1. Qu	3. Qu	*N*	Median	1. Qu	3. Qu	*N*	

PRM	8.55*	2.17	34.22	14	8.09*	0.54	23.13	14	0.279
CBZ	12.08*	0.00	31.05	14	9.21*	0.35	18.37	14	0.279
LEV	12.22*	1.96	30.85	14	7.61*	0.57	25.97	14	0.683
LTG	3.91*	0.22	14.25	14	0.94*	0.00	6.42	14	0.003
VPA	2.36*	0.45	9.20	14	1.70*	0.08	10.07	14	0.480
OXC	6.77*	0.04	29.97	14	2.56*	0.21	21.91	14	0.025
TPM	10.99*	1.68	43.71	14	18.98	3.56	66.84	14	0.177
PB	10.06*	0.12	35.27	14	6.53*	0.08	32.58	14	0.374
Lithium	24.63	2.34	56.06	14	24.40	5.38	92.58	14	0.140

	IL-2 (pg/mL)								
	Median	1. Qu	3. Qu	*N*					

Unstimulated blood	0.24	0.00	1.97	14					
OKT3/5C3-stimulated blood	18.26^†^	2.59	88.08	13					

Concentration	1-fold				2-fold				Sig.
	Median	1. Qu	3. Qu	*N*	Median	1. Qu	3. Qu	*N*	

PRM	12.82*	2.70	28.99	14	11.66*	2.90	34.05	14	0.463
CBZ	7.61*	1.42	38.15	14	8.28*	2.17	17.24	14	0.507
LEV	9.74*	3.46	31.38	14	5.22*	1.42	23.64	14	0.055
LTG	3.58*	0.80	15.90	14	2.89*	0.00	12.82	14	0.050
VPA	4.64*	0.50	20.42	14	3.77*	0.77	41.65	14	0.055
OXC	5.79*	1.04	17.20	14	3.87*	0.05	15.75	14	0.021
TPM	6.06*	2.67	25.69	14	10.61*	4.29	19.32	14	0.917
PB	9.13*	0.80	28.85	14	7.28	2.86	33.85	14	0.279
Lithium	22.95	9.79	61.02	14	104.30*	65.10	233.18	14	0.002

	IL-4 (pg/mL)								
	Median	1. Qu	3. Qu	*N*					

Unstimulated blood	0.02	0.00	0.17	14					
OKT3/5C3-stimulated blood	3.15^†^	0.26	10.78	13					

Concentration	1-fold				2-fold				Sig.
	Median	1. Qu	3. Qu	*N*	Median	1. Qu	3. Qu	*N*	

PRM	2.83	0.36	9.93	14	2.47	0.40	8.50	14	0.046
CBZ	1.83	0.21	9.43	14	1.13*	0.08	8.43	14	0.158
LEV	2.56	0.62	9.64	14	1.79	0.14	6.89	14	0.010
LTG	0.50*	0.00	8.15	14	0.44*	0.05	2.39	14	0.110
VPA	0.00*	0.00	1.76	14	0.00*	0.00	2.60	14	0.866
OXC	0.79	0.05	10.23	14	0.40	0.05	9.56	14	0.209
TPM	2.10	0.23	12.70	14	2.03	0.56	10.33	14	0.485
PB	2.25	0.47	12.15	14	1.28	0.25	10.66	14	0.007
Lithium	4.33	0.89	14.27	14	5.34*	2.46	11.57	14	0.096

	IL-6 (pg/mL)								
	Median	1. Qu	3. Qu	*N*					

Unstimulated blood	0.26	0.02	0.53	14					
OKT3/5C3-stimulated blood	380.80^†^	13.16	865.67	13					

Concentration	1-fold				2-fold				Sig.
	Median	1. Qu	3. Qu	*N*	Median	1. Qu	3. Qu	*N*	

PRM	38.10*	11.03	299.12	14	59.29	3.23	185.31	14	0.300
CBZ	50.13*	4.57	238.95	14	72.53*	16.07	312.94	14	0.972
LEV	73.82*	32.84	206.15	14	56.35*	22.34	267.09	14	0.925
LTG	23.08*	1.63	122.41	14	5.03*	0.82	31.41	14	0.004
VPA	9.36*	1.23	22.32	14	1.46*	0.73	11.38	14	0.177
OXC	27.61*	1.33	165.59	14	7.73*	0.79	65.89	14	0.026
TPM	87.06*	17.34	194.20	14	88.07*	30.94	190.05	14	0.551
PB	75.39*	1.08	226.22	14	53.65	8.98	365.82	14	0.925
Lithium	244.70	20.98	811.36	14	592.21	149.34	1958.24	14	0.016

	IL-17 (pg/mL)								
	Median	1. Qu	3. Qu	*N*					

Unstimulated blood	0.00	0.00	0.33	14					
OKT3/5C3-stimulated blood	6.99^†^	1.19	44.19	13					

Concentration	1-fold				2-fold				Sig.
	Median	1. Qu	3. Qu	*N*	Median	1. Qu	3. Qu	*N*	

PRM	8.28	1.14	27.17	14	5.76	1.11	21.36	14	0.039
CBZ	6.95	0.08	26.99	14	4.73*	0.00	27.68	14	0.158
LEV	7.42	0.69	29.47	14	2.98	0.61	29.35	14	0.530
LTG	4.49*	0.00	12.67	14	1.53*	0.06	3.78	14	0.041
VPA	1.08*	0.00	3.36	14	0.70*	0.00	4.61	14	0.286
OXC	5.10	0.56	14.30	14	3.84*	0.00	10.68	14	0.060
TPM	7.30	1.51	31.82	14	9.84	0.52	26.30	14	0.638
PB	8.90	0.30	22.57	14	5.10*	0.27	23.74	14	0.209
Lithium	15.23*	4.26	69.95	14	17.80*	6.60	71.26	14	0.331

	IL-22 (pg/mL)								
	Median	1. Qu	3. Qu	*N*					

Unstimulated blood	92.00	92.00	92.00	2					
OKT3/5C3-stimulated blood	445.00	278.00	1002.00	7					

Concentration	1-fold				2-fold				Sig.
	Median	1. Qu	3. Qu	*N*	Median	1. Qu	3. Qu	*N*	

PRM	314.00	179.50	733.50	8	273.00	212.00	888.00	7	0.753
CBZ	315.00	231.00	323.00	7	284.50	171.75	1027.75	6	0.753
LEV	246.00	131.50	382.00	8	365.50	263.50	1353.50	6	0.043
LTG	317.00	182.50	1839.75	4	348.00	106.50	1046.00	5	0.068
VPA	374.00	180.00		3	68.00	47.50	749.00	5	0.109
OXC	321.00	128.25	1094.75	6	222.00	126.00	1152.50	5	0.144
TPM	376.00	179.25	1183.25	6	305.00	85.25	1260.75	8	0.893
PB	336.00	266.50	1935.00	5	414.00	315.25	1099.25	6	0.465
Lithium	576.00	335.00	909.50	9	803.00*	312.00	1317.00	11	0.028

	TNF-*α* (pg/mL)								
	Median	1. Qu	3. Qu	*N*					

Unstimulated blood	0.00	0.00	0.28	14					
OKT3/5C3-stimulated blood	92.80^†^	1.80	503.56	13					

Concentration	1-fold				2-fold				Sig.
	Median	1. Qu	3. Qu	*N*	Median	1. Qu	3. Qu	*N*	

PRM	206.87	11.68	540.99	14	167.15	17.03	530.13	14	0.470
CBZ	149.75	12.89	388.72	14	93.72	7.44	307.18	14	0.701
LEV	121.55	21.20	379.89	14	81.81	9.87	452.42	14	0.917
LTG	48.31	2.01	203.86	14	14.90	0.00	76.64	14	0.272
VPA	7.28*	0.72	31.57	14	8.37*	0.60	64.47	14	0.583
OXC	88.64	0.32	352.93	14	83.95	1.21	442.08	14	0.861
TPM	133.71	22.48	542.04	14	240.79	15.32	887.72	14	0.008
PB	99.87	4.69	589.68	14	100.96	4.92	385.65	14	0.754
Lithium	326.25	43.69	912.04	14	364.50*	85.15	1027.79	14	0.019

*N* = number of measurable cytokines. ^†^Significant difference between cytokine values in unstimulated and OKT3/5C3-stimulated blood. *Significant difference between cytokine values in OKT3/5C3-stimulated blood and OKT3/5C3-stimulated blood with supplementation of the listed drugs at specified concentrations. Sig.: level of significance of the difference between cytokine values at the 1-fold and cytokine values at the 2-fold drug concentration.
